# Supplementary material for: Multidimensional predictors of common mental disorders among Indian mothers of 6- to 24-month-old children living in disadvantaged rural villages with women’s self-help groups: A cross-sectional analysis
Source: PLoS One. 2020 Jun 23;15(6):e0233418. doi: 10.1371/journal.pone.0233418 (PMC7310838; doi:10.1371/journal.pone.0233418)
Supplement: S1 Table — (DOCX) [file pone.0233418.s001.docx]

| **Supplemental Table 1. Variable definitions** | |
| --- | --- |
| Variable | Definition |
| **Outcomes** |  |
| SRQ score | Score (0-20) on Self-Reporting Questionnaire^1^. A higher number indicates higher mental distress. |
| Common mental disorder (CMD) symptoms | SRQ score of ≥ 8 out of 20. |
| **Women’s work** |  |
| Primary occupation |  |
| Ag. laborer | Dummy variable = 1 if primary occupation reported as farmer of crops or an agricultural day labourer. Other answer options were: Non-agricultural day labourer, service/salaried worker, small/cottage industry, business/trader, skilled worker, other self-employed, house worker/housewife, migrant labourer, retired/old age, physically challenged, student, not working or other. |
| Housewife | Dummy variable = 1 if primary occupation reported as housewife |
| Other | Dummy variable = 1 if primary occupation reported as non-agricultural day labourer, service/salaried worker, small/cottage industry, business/trader, skilled worker, other self-employed, migrant labourer, retired/old age, physically challenged, student, not working or other. |
| Hours of work per day | Hours of work spent on work as: employed, own business work, staple grain farming, horticultural (gardens) or high value crop farming, large livestock raising (cattle, buffaloes), small livestock raising (sheep, goats, pigs), poultry and other small animal raising, fishpond culture, commuting (to/from work or school), shopping, getting service (incl. health services), weaving/sewing/textile care, cooking, domestic work (incl. fetching water and fuel), caring for children, and caring for adults. |
| Proportion of working time spent on HH chores/care | The ratio of time spent on: [shopping, getting service (including health services), weaving/sewing/textile care, cooking, domestic work (including fetching water and fuel), caring for children, and caring for adults] to total hours worked per day. |
| Proportion of working time spent on farm/livestock/employed activities | The ratio of time spent on: [staple grain farming, horticultural (gardens) or high value crop farming, large livestock raising (cattle, buffaloes), small livestock raising (sheep, goats, pigs), poultry and other small animal raising, fishpond culture, work as employed, and own business work] to total hours worked per day. |
| **Women’s agency** |  |
| Decision making score | Respondent’s score 0-1 on whether she, herself, generally makes decisions about: whether she could eat eggs while pregnant or breastfeeding with last child, whether she could consume milk or mild products while pregnant or breastfeeding last child, whether she could eat meat, poultry or fish while pregnant or breastfeeding last child, whether to breastfeed last child, whether to stop breastfeeding last child, whether or not last child is offered eggs to eat, whether or not last child is offered milk or milk products, whether or not last child is offered meat, poultry or fish, when to start introducing foods and liquids (other than breastmilk) to last child, how much she worked while pregnant or breastfeeding last child, how much she could rest while pregnant or breastfeeding last child, and who will care for last child when she needs to go outside the home for an extended period of time. |
| Score of progressive attitudes (higher is more progressive) | Respondent score 0-1 on her progressive opinions, the higher the score the more progressive because she: disagrees with “man should take the important decisions”, agrees with “husband should help in house if women works outside”, disagrees with “husband should not let his wife work outside home”, agrees with “woman has the right to express her opinion”, disagrees with “woman must accept that her husband or partner beats her”, and disagrees with “it is better to send a son to school than a daughter”. |
| Self-help group member | Dummy variable = 1 belongs to a self-help group. |
| **Woman’s own health, nutrition, and reproductive history** |  |
| Woman underweight | Dummy variable = 1 if BMI < 18.5 kg/m^2^. |
| Woman normal weight | Dummy variable = 1 if BMI 18.5-24.9 kg/m^2^ |
| Woman overweight | Dummy variable = 1 if BMI 25-29.9 kg/m^2^ |
| Woman obese | Dummy variable = 1 if BMI ≥ 30 kg/m^2^ |
| Pregnant before age 18 years | Dummy variable = 1 if first pregnancy occurred before 18^th^ birthday. |
| Ever had failed pregnancy | Dummy variable = 1 if number of pregnancies > number of children. |
| Currently pregnant | Dummy variable = 1 if mother is currently pregnant. |
| Woman achieved minimum dietary diversity | Dummy variable = 1 if mother consumed at least 5 of 10 food groups on the previous day. Food groups included staple cereals, pulses, nuts and seeds, dairy, flesh foods (meat or fish), vitamin A rich fruits and vegetables, green leafy vegetables, other fruits, other vegetables. |
| **Child age and health** |  |
| Child age | Child age in months |
| Child female | Dummy variable = 1 if child is female. |
| Child aged 6-11 months | Dummy variable = 1 if child is 6-11 months old and = 0 if child is 12-24 months old. |
| Child sick in last 2 weeks | Dummy variable = 1 if child had either fever, cough/cold, fast/short breathing or diarrhea in the last 2 weeks. |
| **Household social status, poverty and wealth** |  |
| Caste |  |
| SC | Dummy variable = 1 if household head is scheduled caste. |
| ST | Dummy variable = 1 if household head is scheduled tribe. |
| OBC | Dummy variable = 1 if household head is OBC. |
| General | Dummy variable = 1 if household head is general caste. |
| Food insecurity scale score | Score (0-27) measured using the Household Food Insecurity Access Scale, where a higher score indicates higher food insecurity^2^. |
| Food secure | Dummy variable = 1 if household is food secure^2^. |
| Mild food insecurity | Dummy variable = 1 if household is mildly food insecure^2^. |
| Moderate food insecurity | Dummy variable = 1 if household is moderately food insecure^2^. |
| Severe food insecurity | Dummy variable = 1 if household is severely food insecure^2^. |
| HH experiences any food insecurity | Dummy variable = 1 if household experiences any food insecurity^2^. |
| Improved drinking water source | Dummy variable =1 if household’s main source of water is either piped water into dwelling, piped water into yard/plot, public tap/standpipe, tube well/borehole or protected spring |
| Improved toilet at household | Dummy variable = 1 if household has a toilet or a pit and does not practice open defecation. |
| Poorest wealth quintile | Household belongs to the poorest quintile of the wealth index. The wealth index was computed using principal component analysis of wealth indicators including mattress, pressure cooker, chair, cot/bed, bed net, table, electric fan, radio, television, sewing machine, mobile phone, landline phone, computer, refrigerator, air conditioner, washing machine, clock or watch, car, motorcycle, bicycle, tractor, water pump, thresher, animal-drawn cart, auto rickshaw, electricity, house building material |
| **Shocks experienced in past year** |  |
| Shock - demonetization | Dummy variable = 1 if household was affected by loss of savings, daily wages, or other loss due to demonetization (government ban on 500 and 1000 notes) in the last 12 months. |
| Shock - death | Dummy variable = 1 if household was affected by death of income earning household member or death of other household member in the last 12 months. |
| Shock - illness | Dummy variable = 1 if household was affected by disease/injury of income-earning household member or disease/injury of other HH member. |
| Shock – non-farm livelihood | Dummy variable = 1 if household was affected by loss of employment of any household member or business failure. |
| Shock – crop loss | Dummy variable = 1 if household was affected by loss of crop due to flooding, drought, plant disease, insects, animals, theft, etc. |
| Shock - livestock | Dummy variable = 1 if household was affected by loss of cattle/livestock/small livestock/poultry due to disease, injury, etc. |
| **Other factors** |  |
| Woman’s age | Mother’s age in years. |
| Woman’s education | Years of schooling finished by mother. |
| Household size | Number of household members. |
| Dependency ratio | Ratio of household members < 16 years or >55 years old to household members 16-55 years old. |
| Interviewer sex | Dummy variable = 1 if interviewer was female. |
| In Nutrition-Intensive PRADAN arm | Dummy variable = 1 if household in nutrition intervention arm (all other households are in Standard PRADAN arm) |
| ^1^ World Health Organization (1994). A user’s guide to self-reporting questionnaires. Geneva, Switzerland.  ^2^ Coates, J., Swindale, A., & Bilinsky, P. (2007). Household food insecurity access scale (HFIAS) for measurement of food access: indicator guide version 3. | |
