# Supplementary material for: Multidimensional predictors of common mental disorders among Indian mothers of 6- to 24-month-old children living in disadvantaged rural villages with women’s self-help groups: A cross-sectional analysis
Source: PLoS One. 2020 Jun 23;15(6):e0233418. doi: 10.1371/journal.pone.0233418 (PMC7310838; doi:10.1371/journal.pone.0233418)
Supplement: S2 Table — (DOCX) [file pone.0233418.s002.docx]

| **Supplemental Table 2. Characteristics of Indian women with children aged 6-24 months by main occupation** | | |
| --- | --- | --- |
|  | **Housewife** | **Farmer** |
|  | n=977 | n=501 |
|  | *Mean (SD)/%* | *Mean (SD)/%* |
| **Outcomes** |  |  |
| SRQ score, 0-20 | 3.9 (3.7) | 3.2 (3.8) |
| CMD, SRQ ≥ 8 | 19 | 10 |
| **Women's work** |  |  |
| Work time per day, hours | 10.3 (2.3) | 11.0 (2.1) |
| HH chores/care, proportion of work time | 88 | 72 |
| Labor-related work, proportion of work time | 12 | 28 |
| **Women's agency** |  |  |
| Decision making score, 0-1^2^ | 0.8 (0.3) | 0.8 (0.4) |
| Progressive gender attitudes score, 0-1^2^ | 0.8 (0.2) | 0.7 (0.2) |
| Self-help group member, % | 43 | 42 |
| **Woman’s own health, nutrition, and reproductive history** |  |  |
| Woman’s nutritional status^3,4^ |  |  |
| Underweight, % | 41 | 36 |
| Normal weight, % | 56 | 57 |
| Overweight, % | 2 | 7 |
| Obese, % | 0 | 0 |
| Woman achieved minimum dietary diversity, % | 31 | 27 |
| Pregnant before age 18 years, % | 13 | 12 |
| Ever had failed pregnancy, % | 28 | 29 |
| Currently pregnant, % | 9 | 8 |
| **Child age and health** |  |  |
| Child less than 1 year old, % | 32 | 29 |
| Child age, months | 14.7 (5.0) | 15.0 (4.9) |
| Child female, % | 46 | 47 |
| Child sick in last 2 weeks, %^5^ | 28 | 30 |
| **Household social status, poverty and health** |  |  |
| Caste |  |  |
| SC, % | 14 | 9 |
| ST, % | 55 | 75 |
| OBC, % | 28 | 14 |
| General, % | 3 | 2 |
| Wealth PCA^7^ | 0.2 (2.3) | -0.54 (1.8) |
| Poorest wealth quintile, % | 17 | 23 |
| Food insecurity scale, 0-27^8^ | 2.2 (3.1) | 3.0 (3.6) |
| Food security^8^ |  |  |
| Food secure, % | 55 | 42 |
| Mild food insecurity, % | 18 | 24 |
| Moderate food insecurity, % | 21 | 29 |
| Severe food insecurity, % | 6 | 6 |
| Water and sanitation |  |  |
| Improved drinking water source, % | 69 | 66 |
| Improved toilet at household, % | 38 | 29 |
| **Shocks experienced in pats year** |  |  |
| Negative shock in last year |  |  |
| Death, % | 6 | 6 |
| Illness, % | 23 | 24 |
| Demonetization, % | 21 | 14 |
| Non-farm livelihoods, % | 6 | 2 |
| Crop loss, % | 29 | 35 |
| Livestock loss, % | 17 | 2 |
| **Individual and household demographics** |  |  |
| Woman’s age, years | 25.2 (4.3) | 25.9 (4.7) |
| Woman’s education, years | 5.2 (4.4) | 4.58 (4.1) |
| Household size, persons | 5.5 (1.8) | 5.5 (1.7) |
| Dependency ratio^6^ | 1.1 (0.7) | 1.13 (0.7) |
| Female interviewer, % | 95 | 99 |
| Nutrition intensive arm, % | 47 | 50 |
| 1. Other occupations included: Non-agricultural day laborer (5.9 %), service/salaried worker (2.2%), migrant laborer (0.4%), business/traders (0.3%) and small/cottage industry (0.2%), 2. Decision making score and progressive gender attitudes were based on 12 and 6 questions respectively; both scores were rescaled from 0 to 1 3. Standard World Health Organization categorization was used for underweight (BMI<18.5 kg/m^2^), normal weight (BMI 18.5-24.9 kg/m^2^), overweight (BMI 25-29.9 kg/m^2^) 4. The sample size with anthropometric measures was slightly smaller (n=1,412 overall; n=1,206 low CMD; n=206 high CMD) 5. Coded as positive if mother reported that child suffered from cough, fever or diarrhea in the previous 2 weeks 6. Ratio of individuals aged <15yrs or >55yrs to those aged 16-55yrs 7. Principal components analysis of mattress, pressure cooker, chair, cot/bed, bed net, table, electric fan, radio, television, sewing machine, mobile phone, landline phone, computer, refrigerator, air conditioner, washing machine, clock or watch, car, motorcycle, bicycle, tractor, water pump, thresher, animal-drawn cart, auto rickshaw, electricity, house building material 8. Measured using the Household Food Insecurity Access Scale (HFIAS), scaled from 0 to 27 with levels of food security defined according to USAID’s FANTA III HFIAS guide (2007).   List of abbreviations: CMD, common mental disorders; OBC, other backward class; SC, scheduled caste; ST, scheduled tribe; SRQ, self-reporting questionnaire | | |
|  |  |  |
|  |  |  |
|  |  |  |
|  |  |  |
