# Supplementary material for: Multidimensional predictors of common mental disorders among Indian mothers of 6- to 24-month-old children living in disadvantaged rural villages with women’s self-help groups: A cross-sectional analysis
Source: PLoS One. 2020 Jun 23;15(6):e0233418. doi: 10.1371/journal.pone.0233418 (PMC7310838; doi:10.1371/journal.pone.0233418)
Supplement: S3 Table — (DOCX) [file pone.0233418.s003.docx]

| **Supplemental Table 3. SRQ scores for SHG members by absence and presence of credit activities in SHG** | | | |  |
| --- | --- | --- | --- | --- |
|  | **Overall**  **women in SHG** | **SHG with no credit activities** | **SHG with credit activities** | **Group difference*** |
|  | n=713 | n=88 | n=625 |  |
|  | *Mean(SD)/%* | *Mean(SD)/%* | *Mean(SD)/%* | *P value* |
| **Outcome** |  |  |  |  |
| SRQ score | 3.6 (3.8) | 3.8 (3.4) | 3.44 (3.7) | 0.40 |
| CMD (SRQ>=8) | 16 | 17 | 14 | 0.39 |

* P-values reported are from t-tests for continuous variables, and from chi2 tests for binary variables
