# Supplementary material for: Multidimensional predictors of common mental disorders among Indian mothers of 6- to 24-month-old children living in disadvantaged rural villages with women’s self-help groups: A cross-sectional analysis
Source: PLoS One. 2020 Jun 23;15(6):e0233418. doi: 10.1371/journal.pone.0233418 (PMC7310838; doi:10.1371/journal.pone.0233418)
Supplement: Attachment — Submitted filename: review_Plos one Dec27.2019.docx [file pone.0233418.s005.docx]

PLOS ONE

Multi-dimensional predictors of common mental disorders among Indian care-givers of 6 to 24-month-old children living in villages with women’s self-help groups.

Thank you very much for giving me an opportunity to review this paper. This study is very important focused on rural area of India and this sample population was quite big. Overall, this paper is well-written and worth publishing.

Here are my comments. Hope these are helpful to improve consistency and persuasiveness.

**Title**

Please include targeted population (living in disadvantaged area, in rural villages)and study design (cross-sectional study).

The participants included those without self-help groups (Table 1), hence it should be deleted.

**Introduction**

1. 1^st^ Paragraph: Authors need to describe more about the impact of CMD of care givers in low and middle income countries or Indian cultural contexts. For example, malnourishment of baby or infanticide?
2. 1^st^ Paragraph: In order to address the importance of mental health issued among caregivers, please add the findings of previous studies with regard to the prevalence of CMD or depression among mothers.
3. I wonder if ‘care giver’ includes father, mother and other relatives? If survey assessed mother, please change the term as mother.
4. 2^nd^ Paragraph, please describe more in detail about determinants of CMD in previous studies. Although third paragraph mentioned the literature regarding social factor for women’s lives is scarce, previous studies have examined cultural factors in 2^nd^ paragraph.
5. What is the originality of this study compared with previous studies? Including variable regarding self-help group? What hypothesis can be considered? Attending in self-help group is one of independent factors?
6. Aim: What brought authors to investigate targeted to caregivers of *young children living in disadvantages areas of rural area,* which reason should be explained in introduction and specified in title and abstract.

**Methods**

1. Although authors selected disadvantageous areas in aims, how did they select them ? Please describe how they selected these states and villages.
2. Exploratory factor analysis: because the SRQ is dichotomous, factor analysis cannot be done.

I wonder if authors conducted factor analysis with categorical data? If not, my suggestion is that authors only remain the result of Chronbach’s alpha value not results of factor analysis.

*Statistical analysis*:

1. Some variables are categorized (Table 1) examined chi2 in order to assess the group differences.

Results

2. Because of the cross-sectional study, it is not sure if authors can use the term ‘protective factor,’ ‘Risk factor’ and ‘predictors.’

Discussion

1. 301-302 In case results are not shown, it should not be discussed because this is academic paper.

Thank you.
