# Supplementary material for: Multidimensional predictors of common mental disorders among Indian mothers of 6- to 24-month-old children living in disadvantaged rural villages with women’s self-help groups: A cross-sectional analysis
Source: PLoS One. 2020 Jun 23;15(6):e0233418. doi: 10.1371/journal.pone.0233418 (PMC7310838; doi:10.1371/journal.pone.0233418)
Supplement: Attachment — Submitted filename: PONE-D review - Dec 19.docx [file pone.0233418.s006.docx]

**Psychosocial determinants of sustained maternal functional impairment: longitudinal findings from a pregnancy-birth cohort study in rural Pakistan**

PONE-D-19-02072R1

Mothers’ quality of life during pregnancy as well as during postnatal period is very important both for their own health and well-being as well as for the child health and development. The authors in this paper have looked at a very important area of identifying the risk and protective factors of mothers’ functional trajectories. This is an excellent manuscript and the authors have addressed the comments from the reviewers I have only some minor points given below.

In abstract authors provided background information, aims, analysis procedure and results. There is no information about methodology. Authors can briefly mention the study setting, outcome measures, sample size.

In “introduction” section authors clearly explained why a mother functioning during postnatal period is important by highlighting the perinatal period as sensitive period for mortality and morbidity. Role of several psychosocial factors have been highlighted such as stress, depression and social support. However, authors did not talk about other factors that can be particularly relevant to mothers in LMICs such as financial difficulties and interpersonal conflicts.

Authors labeled group 1 as women persistently well-functioning, group 2 poor functioning with modest recovery, and group 3 as chronically poor functioning. It is clear that these categories came from WHODAS, however it will be helpful if the authors could give operational definitions for the three categories.
