# Supplementary material for: Multidimensional predictors of common mental disorders among Indian mothers of 6- to 24-month-old children living in disadvantaged rural villages with women’s self-help groups: A cross-sectional analysis
Source: PLoS One. 2020 Jun 23;15(6):e0233418. doi: 10.1371/journal.pone.0233418 (PMC7310838; doi:10.1371/journal.pone.0233418)
Supplement: Attachment — Submitted filename: PLOS review - NH.docx [file pone.0233418.s007.docx]

**Multidimensional predictors of common mental disorders among Indian caregivers of 6- to 24-month-old children living in villages with women’s self-help groups**

**PONE-D-19-27808**

Poor mental health of caregivers of young children and its impact on their own as well as children’s wellbeing are now well established including some interesting findings from low and middle income countries. However it is important to understand the protective and risk factors for poor mental health of caregivers which can inform development of culturally relevant interventions for this target population.

In introduction section, Authors mentioned consequences of CMD among caregivers in terms of loss of productivity and impact on child growth and development. Though authors talked about limited health and wellbeing budget in India and also discussed women self-help groups (SHG), however, there is no information on risk factors of CMD for caregivers of young children in low resource settings such as India. Some information on content of self-help groups would be useful. Authors also need to talk about what other support programs/models exist to support mothers or caregivers of young children in Indian context. The reference mentioned on line number 53, page number 10 for relationship between CMD among caregivers and loss of productivity is not specifically for the caregivers. This is for adults with chronic medical conditions. A very similar study was published in 2011 in Korea (Bang, K.S., Chae, S.M. and Park, S.H., 2011. Depression, health status, and parenting stress of caregivers of children in poverty. *Korean Parent-Child Health Journal*, *14*(2), pp.55-61.)

To my understanding all the caregivers were “mothers” therefore I was wonderinng why authors used the term caregivers rather than mothers. In method section authors explained the ethical considerations such as voluntary participation and ethics approval from a review board. It would be helpful to mention use of participant information sheets in local languages and how the consent was obtained from participants with limited reading and writing skills. Not clear why the researchers obtained verbal consent? Why not written consent such as thumb print? Researchers are also now using audio recorded consents.

While describing household and environmental indicators, authors mentioned caste categories and they used term “other backward classes” (OBC) on line number 163. The term seems very stigmatizing. Can we think of an alternative name that can describe this cast category. There is no information on who approached the participants, who did assessments, training and supervision of assessors. This information is important to understand the quality of the data collected.

In discussion section authors discussed difference between farmers and housewives – being farmer would be a protective factor. This needs to be discussed further. As mixed evidence exist such as research saying no association between farming and increased mental health problems (Judd et al., 2006). On other hand evidence also exist to support high rates of suicide among farmers (Sainath. P ‘Farmers suicide rates soar above the rest’. The Hindu, May 18, 2013, Mumbai).

In limitations section on page 21, 342 – 352, authors need to mention use of a self-reporting questionnaire to assess depression rather than a structured interview is also the limitation of the study. Moreover, while talking about the components/variables that authors could not assess, they also need to talk about domestic violence, possible significant contributor of poor mental health in women and especially in mothers of young children.
