# Supplementary material for: Multidimensional predictors of common mental disorders among Indian mothers of 6- to 24-month-old children living in disadvantaged rural villages with women’s self-help groups: A cross-sectional analysis
Source: PLoS One. 2020 Jun 23;15(6):e0233418. doi: 10.1371/journal.pone.0233418 (PMC7310838; doi:10.1371/journal.pone.0233418)
Supplement: Attachment — Submitted filename: WINGS_mental health_responses to reviewers_final.docx [file pone.0233418.s008.docx]

| Comment | Response |
| --- | --- |
| Reviewer 1 |  |
| Thank you very much for giving me an opportunity to review this paper. This study is very important focused on rural area of India and this sample population was quite big. Overall, this paper is well-written and worth publishing.  Here are my comments. Hope these are helpful to improve consistency and persuasiveness. | We appreciate your feedback. Please see our responses to your comments below. |
| **Title**  Please include targeted population (living in disadvantaged area, in rural villages) and study design (cross-sectional study).  The participants included those without self-help groups (Table 1), hence it should be deleted. | We have added the targeted population and study design to the title.  We feel it is important to note that the study setting was rural villages where women’s self-help groups are actively being promoted by an NGO (PRADAN), particularly given the key finding that group membership was associated with better mental health. We also want the paper to reach multiple audiences, including those interested specifically in women’s self-help groups. The findings on group membership would also not be valid if the population being studied consisted only of SHG members.  The new proposed title is “Multidimensional predictors of common mental disorders among Indian mothers of 6- to 24-month-old children living in disadvantaged rural villages with women’s self-help groups: a cross-sectional analysis”. |
| **Introduction**   1. 1^st^ Paragraph: Authors need to describe more about the impact of CMD of care givers in low and middle income countries or Indian cultural contexts. For example, malnourishment of baby or infanticide? | We have mentioned that “Depression is the leading cause of disability worldwide (World Health Organization, 2018) and CMD among mothers have been linked to lost productivity (Egede, 2007), increased healthcare expenses (Soni et al., 2016), poor physical and cognitive development in children (Nguyen et al., 2018), and high long-term costs (Smith Fawzi et al., 2019)” which covers child nutrition and studies in India. Moreover, we are looking at common mental disorders, not severe mental illness, which is outside the scope of our data collection efforts and cannot be analyzed using our data. |
| 2. 1^st^ Paragraph: In order to address the importance of mental health issued among caregivers, please add the findings of previous studies with regard to the prevalence of CMD or depression among mothers. | We have added the prevalence of postpartum depression in India from a systematic review and meta-analysis of 20 studies (Upadhyay et al. 2017). Beyond the postpartum period (0-6 weeks after birth), the prevalence of CMD in mothers is not well described. |
| 3. I wonder if ‘care giver’ includes father, mother and other relatives? If survey assessed mother, please change the term as mother. | All respondents were mothers. We have changed caregiver to mother throughout the paper. |
| 4. 2^nd^ Paragraph, please describe more in detail about determinants of CMD in previous studies. Although third paragraph mentioned the literature regarding social factor for women’s lives is scarce, previous studies have examined cultural factors in 2^nd^ paragraph. | We have expanded the description of the determinants of CMD. We have added a more detailed description of the mechanism underlying the association between maternal CMD and child development, with a focus on low-income contexts, citing an additional study (Herba et al. 2016). We have revised the statement in the 3^rd^ paragraph to resolve the discrepancy noted, citing an additional study which reports on determinants of psychological distress in Indian mothers (Prost et al. 2012). |
| 5. What is the originality of this study compared with previous studies? Including variable regarding self-help group? What hypothesis can be considered? Attending in self-help group is one of independent factors? | The originality of this study lies in 1) the breadth of included measures spanning multiple aspects of women’s lives that may relate to mental wellbeing (women’s work/time, agency/decision-making, group membership, health and nutrition, household, environment, demographics); very few studies take such a holistic approach; 2) collecting these measures on women with young children in a particular context: villages with women’s SHGs – given the rapid increase in women’s SHGs in India (as part of the Government of India’s National Rural Livelihoods Mission program, along with non-governmental efforts such as PRADAN) and the use of this platform for various livelihood and wellbeing interventions, it is important to understand the relationship between SHG membership and CMD. This point has been expanded on in the abstract. Very few studies have examined this relationship. It is also important to separate the effect of group membership from the effect of interventions delivered to groups. We have added this point to the discussion with an additional reference of an earlier study which found an impact of a group-based intervention but did not examine/report the effect of group membership (Tripathy et al. 2010). |
| 6. Aim: What brought authors to investigate targeted to caregivers of *young children living in disadvantages areas of rural area,* which reason should be explained in introduction and specified in title and abstract. | Women are usually targeted by SHG interventions, but most studies of nutritional outcomes focus on the children, not the mother. We chose this topic because SHGs aim to improve women’s well-being and mental health is an important aspect of well-being. |
| **Methods**   1. Although authors selected disadvantageous areas in aims, how did they select them ? Please describe how they selected these states and villages. | The current investigation was a sub-study within a larger evaluation of PRADAN/PHRN’s nutrition behavior change intervention delivered through SHGs. The cross-sectional survey of women with young children was added halfway through the evaluation to better examine impacts of the intervention on maternal/child outcomes, as the household sample of the larger evaluation did not include a sufficient number of mothers with young children. The study setting was villages where the PRADAN/PHRN intervention was active; the states and villages were selected by PRADAN based on many years of SHG promotion. We have added details to describe the study setting in the title and abstract. The ‘Setting and study design’ section in the Methods frames the current analysis in lieu of the larger evaluation. |
| 2. Exploratory factor analysis: because the SRQ is dichotomous, factor analysis cannot be done.   I wonder if authors conducted factor analysis with categorical data? If not, my suggestion is that authors only remain the result of Chronbach’s alpha value not results of factor analysis. | The SRQ is a 20-item scale with all yes/no items, so is scored from 0 to 20. We performed factor analysis using the score from 0 to 20 and use this score as a continuous outcome in multivariate ordinary least squares regression analysis (Table 2). Separately, we generate a binary outcome (<8 of 20 vs. 8 or more of 20) of CMD to use in logistic regression to estimate the odds of CMD associated with various factors. |
| *Statistical analysis*:   1. Some variables are categorized (Table 1) examined chi2 in order to assess the group differences. | We have now used chi2 test for binary variables and t-tests for continuous variables. The p values in Table 1 have been updated accordingly. None of the inferences have changed. |
| Results   2. Because of the cross-sectional study, it is not sure if authors can use the term ‘protective factor,’ ‘Risk factor’ and ‘predictors.’ | We have modified the language of protective and risk factors to ‘factors associated with’. Predictors is only used in a statistical sense to denote right hand side variables. |
| Discussion   1. 301-302 In case results are not shown, it should not be discussed because this is academic paper. | We have created a supplemental table to show the results. Please see Supplementary Table 3. We defer to the editor whether to include this or not. |
| Reviewer 2 |  |
| Poor mental health of caregivers of young children and its impact on their own as well as children’s wellbeing are now well established including some interesting findings from low and middle income countries. However it is important to understand the protective and risk factors for poor mental health of caregivers which can inform development of culturally relevant interventions for this target population | We appreciate your feedback. Please see our responses to your comments below. |
| In introduction section, Authors mentioned consequences of CMD among caregivers in terms of loss of productivity and impact on child growth and development. Though authors talked about limited health and wellbeing budget in India and also discussed women self-help groups (SHG), however, there is no information on risk factors of CMD for caregivers of young children in low resource settings such as India. Some information on content of self-help groups would be useful. | We have expanded the description of the determinants of CMD. We have added a more detailed description of the mechanism underlying the association between maternal CMD and child development, with a focus on low-income contexts, citing an additional study (Herba et al. 2016). We have also cited a study which reports on determinants of psychological distress in Indian mothers (Prost et al. 2012). The content of SHG discussions varies according to the issues that members/community face, but often relates to their economic livelihoods, family, community, health, etc. We have added this information to the introduction. |
| Authors also need to talk about what other support programs/models exist to support mothers or caregivers of young children in Indian context. | We agree with the reviewer that this is an important point that should be emphasized. SHGs and SHG-based interventions are a particularly promising approach for addressing maternal CMD since mothers in these villages have extremely limited access to mental health services. Given patterns of village exogamy, mothers could also be isolated from social support networks. We have added to the introduction, along with supporting references (Shankardass, 2018, Rosenzweig & Stark, 1989). We have also come back to this in the final paragraph of the paper. |
| The reference mentioned on line number 53, page number 10 for relationship between CMD among caregivers and loss of productivity is not specifically for the caregivers. This is for adults with chronic medical conditions. A very similar study was published in 2011 in Korea (Bang, K.S., Chae, S.M. and Park, S.H., 2011. Depression, health status, and parenting stress of caregivers of children in poverty. *Korean Parent-Child Health Journal*, *14*(2), pp.55-61.) | We have revised the sentence to indicate that this relationship (CMD and productivity) is not specifically for caregivers in the Egede paper. Thank you for the reference, but the contexts of Korea and rural India are very different and the article is only available in Korean. |
| To my understanding all the caregivers were “mothers” therefore I was wonderinng why authors used the term caregivers rather than mothers. | All respondents were mothers. We have changed caregiver to mother throughout the paper. |
| In method section authors explained the ethical considerations such as voluntary participation and ethics approval from a review board. It would be helpful to mention use of participant information sheets in local languages and how the consent was obtained from participants with limited reading and writing skills. Not clear why the researchers obtained verbal consent? Why not written consent such as thumb print? Researchers are also now using audio recorded consents. | Thank you for pointing this out. Written consent was obtained. This mistake has been corrected. |
| While describing household and environmental indicators, authors mentioned caste categories and they used term “other backward classes” (OBC) on line number 163. The term seems very stigmatizing. Can we think of an alternative name that can describe this cast category. | We recognize that OBC is stigmatizing, but it is an official classification of the Government of India. |
| There is no information on who approached the participants, who did assessments, training and supervision of assessors. This information is important to understand the quality of the data collected. | We have added details on survey procedures including training, survey team composition, data collection, and monitoring. |
| In discussion section authors discussed difference between farmers and housewives – being farmer would be a protective factor. This needs to be discussed further. As mixed evidence exist such as research saying no association between farming and increased mental health problems (Judd et al., 2006). On other hand evidence also exist to support high rates of suicide among farmers (Sainath. P ‘Farmers suicide rates soar above the rest’. The Hindu, May 18, 2013, Mumbai). | While there is literature highlighting poor mental health of farmers, including recent public attention on high rates of suicide among male Indian farmers in certain Indian states, 1) there is also literature showing that suicide is not more prevalent in agricultural workers than other professions in India (e.g. Patel V. et al. Lancet 2012; the Judd et al. study is for male Australian farmers, which limits comparability in our case); 2) our study is not focused on suicide but rather mild symptoms of depression; 3) it is important to keep in mind the sample and comparison made in our study when comparing to previous studies. Our sample consisted entirely of mothers of young children; we did a simple cross-sectional comparison of mothers who reported their *primary* occupation as housewife (“housewives”) and mothers who reported their *primary* occupation as farmer (“farmers”). We offer a set of plausible explanations for the finding that housewives reported more symptoms of depression than farmers, but our data cannot uncover the (social, political, economic) underpinnings of such a complex association, thus we feel further speculation or references to studies in different populations would not be helpful to the discussion. We are not aware of any studies reporting on mental health in mothers who are housewives vs. mental health in mothers who are engaged in agriculture. |
| In limitations section on page 21, 342 – 352, authors need to mention use of a self-reporting questionnaire to assess depression rather than a structured interview is also the limitation of the study. | We have added this limitation to the discussion but clarify that it is self-reported to an enumerator/interviewer who conducts a face-to-face interview using a structured questionnaire. |
| Moreover, while talking about the components/variables that authors could not assess, they also need to talk about domestic violence, possible significant contributor of poor mental health in women and especially in mothers of young children. | We have added this limitation to the discussion. |
| Reviewer 3 |  |
| Mothers’ quality of life during pregnancy as well as during postnatal period is very important both for their own health and well-being as well as for the child health and development. The authors in this paper have looked at a very important area of identifying the risk and protective factors of mothers’ functional trajectories. This is an excellent manuscript and the authors have addressed the comments from the reviewers I have only some minor points given below. | We appreciate your feedback. Please see our responses to your comments below. |
| In abstract authors provided background information, aims, analysis procedure and results. There is no information about methodology. Authors can briefly mention the study setting, outcome measures, sample size. | The study setting, outcome measures and sample size is already reported in the abstract. |
| In “introduction” section authors clearly explained why a mother functioning during postnatal period is important by highlighting the perinatal period as sensitive period for mortality and morbidity. Role of several psychosocial factors have been highlighted such as stress, depression and social support. However, authors did not talk about other factors that can be particularly relevant to mothers in LMICs such as financial difficulties and interpersonal conflicts. | We have expanded the description of the determinants of CMD. We have added a more detailed description of the mechanism underlying the association between maternal CMD and child development, with a focus on low-income contexts, citing an additional study (Herba et al. 2016). We have also cited a study which reports on determinants of psychological distress in Indian mothers (Prost et al. 2012). |
| Authors labeled group 1 as women persistently well-functioning, group 2 poor functioning with modest recovery, and group 3 as chronically poor functioning. It is clear that these categories came from WHODAS, however it will be helpful if the authors could give operational definitions for the three categories. | Our paper does not include these three categories; the comment is not relevant to our paper. |
