# Supplementary material for: Multidimensional predictors of common mental disorders among Indian mothers of 6- to 24-month-old children living in disadvantaged rural villages with women’s self-help groups: A cross-sectional analysis
Source: PLoS One. 2020 Jun 23;15(6):e0233418. doi: 10.1371/journal.pone.0233418 (PMC7310838; doi:10.1371/journal.pone.0233418)
Supplement: Attachment — Submitted filename: Plos One_review.2020.02.25.docx [file pone.0233418.s009.docx]

Plos One：

Multidimensional predictors of common mental disorders among Indian mothers of 6-

to 24-month-old children living in disadvantaged rural villages with women’s self-help

groups: a cross-sectional analysis

Thank you for reflecting my feedback on your revision.

Here are my comments

1. Title

Although I understand author’s point it is important to highlight self-help group, it is too long.

My suggestion is below

Multidimensional predictors of common mental disorders among Indian mothers of ~~6-~~

~~to 24-month-old children~~ infants and toddlers living in disadvantaged rural villages ~~with women’s self-help groups~~: a cross-sectional analysis

Introduction. 5. Strength

Ideally, these originalities can be described in earlier part such as introduction.

Methods. 2. Exploratory factor analysis

The author’s response does not make sense. Dichotomous scale cannot be applied to (general) exploratory factor analysis statistically. My suggestion is to delete the description of exploratory factor analysis. (Line 150-153, P7)
